# Inbreeding Avoidance Drives Consistent Variation of Fine-Scale Genetic Structure Caused by Dispersal in the Seasonal Mating System of Brandt’s Voles

**DOI:** 10.1371/journal.pone.0058101

**Published:** 2013-03-14

**Authors:** Xiao Hui Liu, Ling Fen Yue, Da Wei Wang, Ning Li, Lin Cong

**Affiliations:** 1 The State Key Laboratory for Biology of Plant Diseases and Insect Pests, Institute of Plant Protection, Chinese Academy of Agricultural Sciences, Beijing, China; 2 Key Laboratory of Weed and Rodent Biology and Management, Institute of Plant Protection, Chinese Academy of Agricultural Sciences, Beijing, China; 3 Institute of Plant Protection, Heilongjiang Academy of Agricultural Sciences, Harbin, Heilongjiang Province, China; Aarhus University, Denmark

## Abstract

Inbreeding depression is a major evolutionary and ecological force influencing population dynamics and the evolution of inbreeding-avoidance traits such as mating systems and dispersal. Mating systems and dispersal are fundamental determinants of population genetic structure. Resolving the relationships among genetic structure, seasonal breeding-related mating systems and dispersal will facilitate our understanding of the evolution of inbreeding avoidance. The goals of this study were as follows: (i) to determine whether females actively avoided mating with relatives in a group-living rodent species, Brandt’s voles (*Lasiopodomys brandtii*), by combined analysis of their mating system, dispersal and genetic structure; and (ii) to analyze the relationships among the variation in fine-genetic structure, inbreeding avoidance, season-dependent mating strategies and individual dispersal. Using both individual- and population-level analyses, we found that the majority of Brandt’s vole groups consisted of close relatives. However, both group-specific FISs, an inbreeding coefficient that expresses the expected percentage rate of homozygosity arising from a given breeding system, and relatedness of mates showed no sign of inbreeding. Using group pedigrees and paternity analysis, we show that the mating system of Brandt’s voles consists of a type of polygyny for males and extra-group polyandry for females, which may decrease inbreeding by increasing the frequency of mating among distantly-related individuals. The consistent variation in within-group relatedness, among-group relatedness and fine-scale genetic structures was mostly due to dispersal, which primarily occurred during the breeding season. Biologically relevant variation in the fine-scale genetic structure suggests that dispersal during the mating season may be a strategy to avoid inbreeding and drive the polygynous and extra-group polyandrous mating system of this species.

## Introduction

Inbreeding depression is a major evolutionary and ecological force influencing population dynamics and the evolution of inbreeding-avoidance traits such as mating systems and dispersal [1]. Population genetic structure is fundamentally determined by mating systems and dispersal, which have long been recognized as be the primary factors influencing the rate and outcome of evolution [2]. Resolving the relationship among genetic structure, mating systems and dispersal will facilitate our understanding of the evolution of inbreeding avoidance.

Inbreeding depression is usually substantial enough to affect both individual and population performance [3], [4] by increasing the chances of offspring being affected by recessive or deleterious traits [5]. Pusey and Wolf (1996) proposed that inbreeding depression is often sufficiently severe to lead to the evolution of inbreeding avoidance mechanisms. In many species, individuals optimize genetic compatibility and avoid costly inbreeding depression by choosing mates based on their genetic relatedness [6]–[10]. The almost ubiquitous phenomenon of females mating with more than one male, polyandry, is proposed as a mechanism to avoid reproducing with genetically incompatible mates [11]–[13] and a means of inbreeding avoidance [14]. The idea that females engage in polyandry for genetic benefits is supported by a great deal of increasingly rigorous empirical evidence [11]–[13], [15]–[24].

The evolutionary causes of dispersal have been the focus of much theoretical work. Kin competition, inbreeding, resource competition and environmental stochasticity have been identified as potential driving forces, but inbreeding avoidance is recognized as one of the primary causes of dispersal [25]. The observations that dispersal occurs more often in one sex or the other [26] and that the relatedness between mates decreases with dispersal distance suggests that dispersal might be an efficient means to avoid inbreeding [27]. Other means of avoiding inbreeding, such as discrimination against kin when choosing a mate, have also evolved [10]. Dispersal and kin discrimination behaviors are inherently connected and should be studied in parallel to better understand their relative effect on the patterns of inbreeding in natural populations [27]–[30].

Mating systems and dispersal are fundamental determinants of population genetic structure [2]. Dispersal can lead to the variations in allele frequencies of local populations, indicated by variation in Fst (Fixation index), a measure of the diversity of randomly chosen alleles within the same sub-population relative to that found in the entire population. The relative fitness of individuals and/or groups may be indirectly indicated by genetic structure, and genetic structure may also be used to predict current and future reproductive success [31]. Fine-scale genetic structure during the breeding season may represent a complex interactive consequence of multiple biological processes [31]. For example, the genetic structure and demography of local populations are tightly linked to the rate and scale of dispersal in the common vole (*Microtus arvalis*) [32]. Patterns of spatial genetic structure may vary during the year because juveniles appear in the population and dispersal occurs [33]. Fine-scale genetic structure in *Malurus cyaneus* is attributed to a high rate of extra-pair paternity and skewed reproductive success among individuals [34]. Genetic assignment methods permit the identification of dispersers in populations [35], and the analysis of genetic structure among populations over time can provide estimates for the effectiveness of dispersal [36]. Thus, resolving the relationship of genetic structure, mating systems and dispersal will allow us to better understand the evolution of inbreeding avoidance.

Brandt’s voles (*Lasiopodomys brandtii*) are non-hibernating herbivorous rodent species that live in complex social groups. These animals are primarily distributed in typical steppe regions in the middle-eastern Inner Mongolia of China, the Republic of Mongolia and the Baikal Lake region of Russia. Continual outbreaks of Brandt’s vole populations have accelerated the erosion and desertification of grasslands, resulting in a heightened focus on this key rodent pest over the last 30 years. Accordingly, the social behavior and mating system of Brandt’s voles have been carefully investigated, including their mate choice patterns, parental care, olfactory communication, spatial distribution and home range [37]–[47]. The results of these studies indicated that the social behaviors of Brandt’s voles are influenced by many factors, including sex, social hierarchy, reproductive status and seasonal environmental factors. However, the mating system of Brandt’s voles has remained controversial, with previous studies postulating systems ranging from promiscuity and polygyny to monogamy. Molecular detection of multiple paternity revealed that Brandt’s vole females are polyandrous in natural populations [48]. Varied individual exchange ratios among groups during the breeding and non-breeding seasons [49] suggest varied dispersal patterns in different seasons.

Brandt’s voles live in groups, each of which occupies a burrow system that is conspicuously visible due to connected burrows and runways [50]. In the wild, the earliest births occurs in March [51], and no over-winter individuals survive through the next August. Thus, in this vole species, no individuals survive for 18 months under the natural environment. Brandt’s voles undergo seasonal reproduction, and the group structure of natural Brandt’s vole populations shows strong periodic seasonal changes. In the non-breeding season, groups are composed of a completely different set of individuals relative to the non-breeding season the year before. At the beginning of the reproductive season, which occurs from approximately March to April in Inner Mongolia, the number of individuals in a group gradually decreases to its lowest level. From about the last 10 days of April to the first 10 days of May, groups are frequently composed of only a male and a female; other group members die or emigrate to a newly recruited breeding group because of mating competition. During the middle and later stages of the reproductive season, which lasts from approximately June to August in Inner Mongolia, the number of individual per group increases to its highest level. In June, groups are frequently composed of a pair of over-winter mates and their progeny, and dispersal becomes common following the sexual maturation of the first generation of the year. Wan reported that males born in April and May can reach sexual maturation at approximately 1.5 months of age, whereas males born in June, July, and August do not reach sexual maturation in the same year. Similarly, females born in April, May, and June can reach sexual maturation at approximately 1 month old and firstly breed at approximately 2 months, whereas females born in July and August do not breed in the same year [52]. The number of litters that a female can produce per year is related to its age: over-winter voles can produce 3–4 litters, and those born in April, May, and June produce 2–3, 1–2, and 0–1 litter in the year of their birth, respectively [52]. At this stage, over-winter individuals begin to die, and the group structure becomes more complex due to dispersal and individual exchanges between groups. Mating behaviors stop around the last 10 days of July and the last progeny are born during the first 10 days of August. September to October is the food-storing stage, a cooperative stage for over-wintering. Exchange of individuals between groups has already been completed, and mating competition has likewise ended [49]. After a peaceful over-winter stage, the new annual reproductive cycle will begin in the spring.

To date, all conclusions about the mating system of Brandt’s voles have been based on observational studies and were primarily conducted under laboratory conditions. No information about the degree of inbreeding or its relationship with the mating system and dispersal of Brandt’s voles in natural populations has been reported. A set of microsatellites recently developed for Brandt’s voles [53], [54] provides a powerful tool to study the group genetic structure and the mating system of this species in depth. The goals of this study were as follows: (i) to determine whether females actively avoided mating with relatives in a group-living rodent species, Brandt’s voles (*Lasiopodomys brandtii*), by combined analysis of mating systems, dispersal and genetic structure; and (ii) to analyze the relationships among variation in fine-genetic structure, inbreeding avoidance, season-dependent mating strategies and individual dispersal.

## Results

### Microsatellite Variation

Characteristics of the vole microsatellite markers are summarized in Table S1. Typing errors and small allele dominance were not detected by MICRO-CHECKER at any of the 14 loci. The number of alleles at the 14 loci ranged from 2 to 21 with an average of 10.64 alleles per locus. The mean polymorphism information content was 0.690, with a range of 0.363 to 0.880. The average values of the observed and the expected heterozygosities were 0.713 (0.480–0.874) and 0.727 (0.477–0.891), respectively. No loci were detected with null alleles for which the frequencies were greater than 0.05. All 14 loci deviated from Hardy–Weinberg equilibrium (HWE) after the Bonferroni correction among some of the 29 sampled groups (ranging from 2 to 8 groups). All but 6 of the 29 groups had at least 1 locus that deviated from HWE (Supporting information, Table S2). We suggest that non-random mating among groups may explain these departures from HWE.

### The Social Composition, Inbreeding Status and Mating System

During the breeding season, only 7 of the 15 groups contained male breeders, and the average across all 15 groups was 0.67 (±0.90) per group (Table 1). Of these, 5 groups contained only 1 male breeder, 1 groups contained 2 male breeders, and 1 groups contained 3 male breeders. In addition, 10 of 15 groups contained female breeders, and the average across all 15 groups was 2.00 (±2.33) per group, ranging from 1 to 8 (Table 1). A paired-samples T-test for the average breeder coefficient [the number of reproductive females (or males)/the number of adult females (or males) in a group] indicated that the proportion of female adults taking part in reproduction was significantly larger than that of males (t = 3.152, df = 13, *P* = 0.008) (Table 1).

**Table 1:** Structure of sampled groups.

Season	Group	The individual number of Group	The number of adults	The number of female adults	The number of male adults	The number of identified male breeders	The male breeder coefficient	Status of male breeders	The number of identified female breeders	The female breeder coefficient	Status of female breeders (the number of embryos of captured pregnant females)
BS	JUN01	10	9	4	5	1	0.20	1	1	0.25	1(9)
BS	JUN02	10	10	3	7	0	0.00	\	1	0.33	1(6)
BS	JUN03	60	21	15	6	2	0.33	1,3	8	0.53	1(12), 2, 3, 4,7, 8(7), 9, 14
BS	JUN04	17	14	6	8	0	0.00	\	2	0.33	1(11), 2(7)
BS	JUN05	24	18	6	12	1	0.08	1	2	0.33	1(10), 2
BS	JUN06	3	2	0	2	0	0.00	\	0	\	\
BS	JUN07	4	4	1	3	0	0.00	\	0	0.00	\
BS	JUN08	5	5	1	4	0	0.00	\	0	0.00	\
BS	JUN12	15	13	6	7	1	0.14	2	6	1.00	1, 2(8), 3(8), 4(8), 5(8), 6(8)
BS	JUN13	20	20	11	9	3	0.33	1, 2, 3	3	0.27	1(6), 2(9), 6(6)
BS	JUN15	17	17	5	12	0	0.00	2	3	0.60	1, 3, 5
BS	JUN17	9	4	3	1	0	0.00	\	0	0.00	\
BS	JUN18	20	14	5	9	1	0.11	1	2	0.40	1, 2(7)
BS	JUN19	8	7	5	2	0	0.00	\	0	0.00	\
BS	JUN20	27	15	6	9	1	0.11	1	2	0.33	1(9), 2
Average			11.53±6.18	5.13±3.83	6.40±3.52	0.67±0.90	0.09±0.12		2.00±2.33	0.31±0.28	
NBS	SEP01	9	9	4	5	0	0.00	\	0	0.00	\
NBS	SEP02	15	15	7	8	0	0.00	\	2	0.29	1, 2
NBS	SEP03	19	19	9	10	3	0.30	4, 6, 8	4	0.44	1, 3, 5, 7
NBS	SEP04	7	7	5	2	0	0.00	\	0	0.00	\
NBS	SEP05	24	24	12	12	3	0.25	6, 9, 10	1	0.08	5
NBS	SEP06	7	7	1	6	0	0.00	\	0	0.00	\
NBS	SEP07	15	15	6	9	0	0.00	\	2	0.33	2, 4
NBS	SEP08	13	13	3	10	0	0.00	\	0	0.00	\
NBS	SEP09	20	20	11	9	0	0.00	\	0	0.00	\
NBS	SEP10	17	17	8	9	2	0.22	1, 4	3	0.38	1, 2, 5
NBS	SEP11	11	11	4	7	0	0.00	\	0	0.00	\
NBS	SEP12	12	12	6	6	0	0.00	\	0	0.00	\
NBS	SEP13	20	20	10	10	0	0.00	\	0	0.00	\
NBS	SEP14	11	11	3	8	0	0.00	\	0	0.00	\
Average			14.29±5.21	6.36±3.30	7.93±2.56	0.57±1.15	0.06±0.11		0.86±1.35	0.11±0.17	

BS: breeding season; NBS: non-breeding season; Status of male (or female) breeders: “1” means the heaviest individual, “2” means the second heaviest individual, “6” means the sixth heaviest individual, and so on.

Group pedigree analysis showed that 51.72% of the groups had no identifiable adult female-male pairs with a close-kin relationship (full sibs or half sibs). This included 7 of 15 groups during the breeding season and 8 of 14 groups during the non-breeding season. This result indicates that approximately half of the sampled groups had no risk of inbreeding. The number of identified adult female-male pairs with a close-kin relationship (full sibs or half sibs) was 65 for the other 8 groups during the breeding season. This was an average of 8.13 (±3.56) pairs per group. During the non-breeding season, there were 51 close-kin pairs in the other 6 groups, with an average of 8.50 (±7.74) pairs per group. If mating had occurred randomly, the potential proportion of inbreeding mates would be 6.38% (the number of close-kin pairs/the sum of possible mates) among the whole population, including all samples collected during the two seasons. Overall, 23 pairs of breeding males and females were identified as mates, and no actual pairing was observed between close relatives, which indicates non-random mating in this species. F_IS_ is an inbreeding coefficient introduced by Wright [55] that expresses the expected rate of homozygosity arising from a given system of breeding, Group specific F_IS_ values showed that none of the 10 groups collected during the breeding season exhibited significant inbreeding (Table 2). Thus, both the results of the group specific F_IS_ analysis and the relationships between identified mates revealed no close-kin mating among these natural Brandt’s vole populations.

**Table 2:** Group specific FIS indices.

Group	The number of juvenile	FIS	P (Rand FIS ≥ Obs FIS)
J01	10	−0.1783	0.9902
J03	58	−0.1217	0.9990
J04	21	−0.0996	0.9296
J05	16	−0.0986	0.9022
J11	6	−0.2903	0.9863
J12	44	−0.2315	1.0000
J13	9	−0.2479	0.9971
J17	5	0.0000	0.5855
J18	13	−0.2897	0.9990
J20	21	−0.3902	1.0000

Overall, 109 embryos from 13 litters (average litter size of 8.38 (±1.66), ranging from 5–12) were used for paternity analysis (Table 3). Paternity analysis indicated multiple paternity in 92% (12/13) of the litters examined. The average number of fathers per litter, including captured fathers and inferred fathers, was 2.38±0.77 and ranged from 1 to 4. Nine litters contained embryos whose fathers were identified among the captured adult males from the group to which the pregnant female belonged. Of these 9 litters, 8 were sired by only 1 captured father, and 1 was sired by 2 captured fathers. Of 10 identified fathers, 6 were the heaviest individuals and 2 were the second heaviest individuals in the group (Table 1). In addition, a subset of embryos from all of these 9 litters were also sired by at least 1 father that escaped capture but was inferred by the genotype of the pregnant females and her embryos. This indicates that these pregnant females mated with at least 1 male not belonging to her group and supports the theory of extra-group polyandry. In total, 22 fathers, including captured fathers and inferred fathers, were identified in these 9 litters through the genotype of the mother and her progeny. Of the 22 fathers that sired these 9 litters, 1 captured father always inseminated the greatest number of embryos in 8 of the 9 litters (Table 3). These results indicate that females preferentially mate with the heaviest male in their own group, but also frequently mate with males outside of their social group. The results of the pedigree analysis showed that the breeding male copulated with all breeding females in a group. For example, all 6 breeding females copulated with only 1 of 7 adult males in group JUN12. This indicates not only polygyny in this species, but also possible hierarchies in the male society of this species.

**Table 3:** Detection of multiple paternity.

Individual	The number of embryosper pregnant female	The total number ofadult males in colony	Captured fathers*	Inferred fathers*	The total number of fathers per set embryo
JUN0105	9	5	2(1+4)	2(1+3)	4
JUN0302	12	6	0	3(2+3+7)	3
JUN0305	7	6	0	2(1+6)	2
JUN0404	11	8	0	3(2+2+7)	3
JUN0524	10	12	1(7)	2(1+2)	3
JUN1207	8	7	1(7)	1(1)	2
JUN1208	9	7	1(2)	1(7)	2
JUN1210	8	7	1(7)	1(1)	2
JUN1213	8	7	1(4)	1(4)	2
JUN1215	8	7	1(7)	1(1)	2
JUN1316	9	9	1(6)	1(3)	2
JUN1817	7	9	1(5)	2(1+1)	3
JUN2013	9	9	0	1(9)	1

The number before the parentheses is the number of captured fathers or inferred fathers. The number in the parentheses following the number of captured fathers or inferred fathers is the number of embryos they have inseminated. For example, “2(1+4)” means that there are two fathers, who inseminated one and four embryos respectively.

### Preferential Dispersal of Males Inferred by Variation in Relatedness

#### A. Within-group relatedness

**Figure 1:**
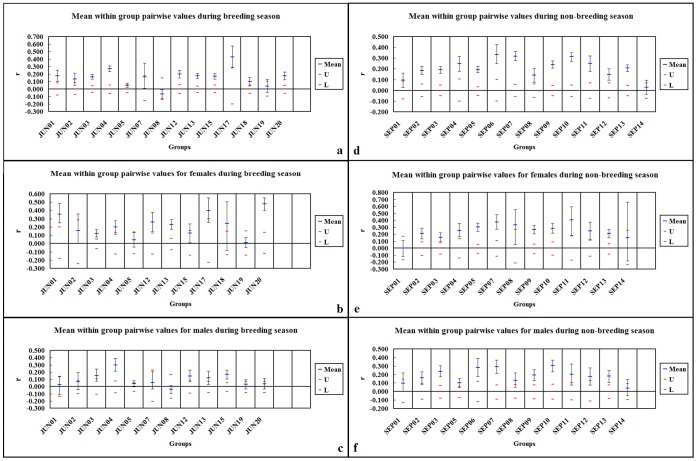
Mean within-group pairwise relatedness estimates. Grey lines represent permuted 95% confidence intervals around the null hypothesis of zero relatedness and error bars represent bootstrapped confidence intervals around the mean. **a,** for the entire group during breeding season; **b,** for the female members of the group during breeding season; **c,** for the male members of the group during breeding season; **d,** for the entire group during non-breeding season; **e,** for the female members of the group during non-breeding season; **f,** for the male members of the group during non-breeding season.

During the breeding season, mean pairwise relatedness within groups was significantly greater than zero in 85.7% (12/14) of groups. Mean pairwise relatedness for females within groups was significantly greater than zero in 75.0% (9/12) of groups. Mean pairwise relatedness for males within groups was significantly greater than zero in 41.7% (5/12) of groups (Fig. 1a –1c). During the non-breeding season, mean pairwise relatedness for females within groups was significantly greater than zero in 84.6% (11/13) of groups, and mean pairwise relatedness for males within groups was significantly greater than zero in 84.6% (11/13) of groups (Fig. 1d –1f). These results indicate that there were more groups comprised of close male relatives during the non-breeding season.

**Figure 2:**
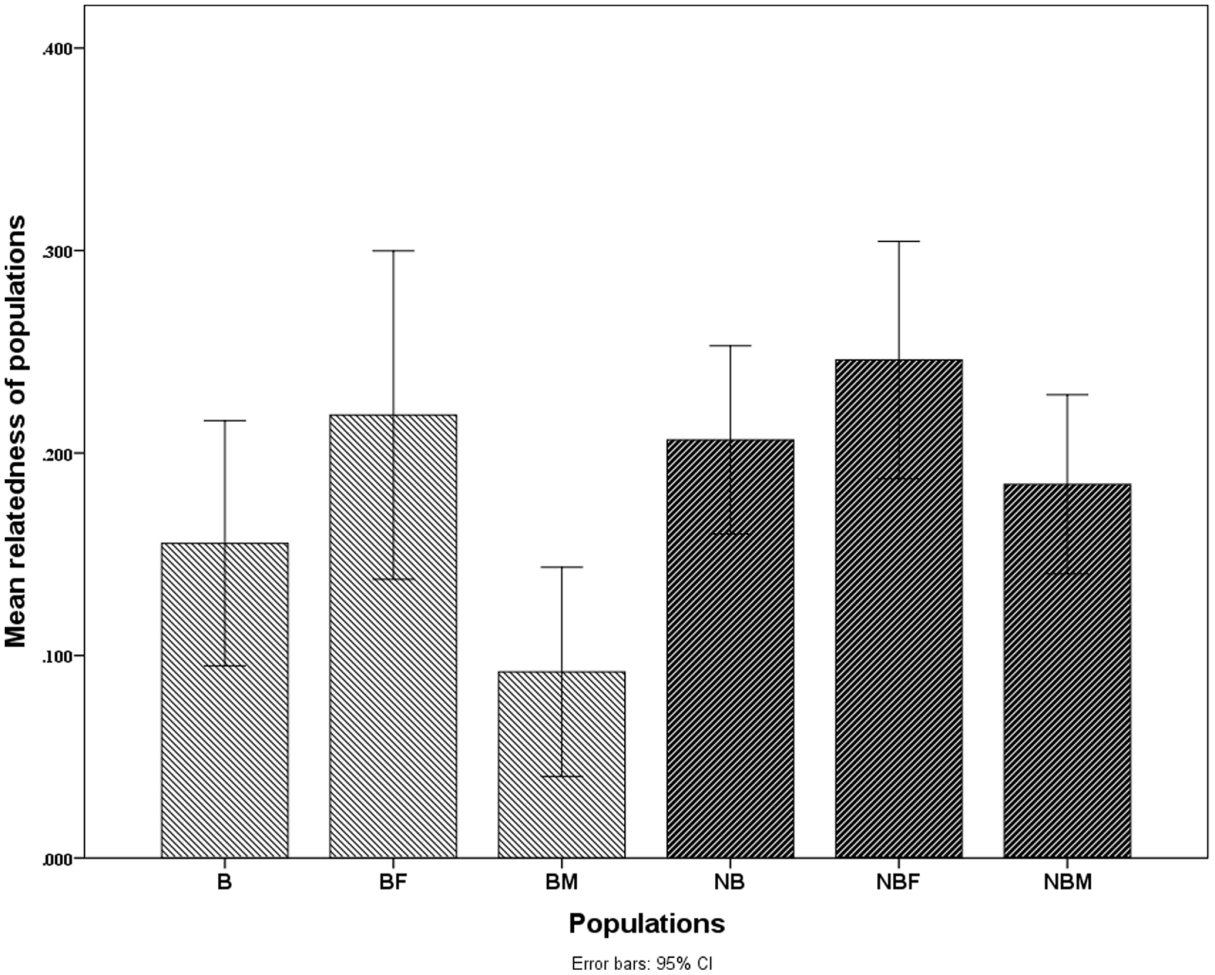
Comparison of within-group pairwise relatedness estimates. **B,** the entire group during breeding season; **NB,** the entire group during non-breeding season; **BF,** the female members of the group during breeding season; **NBF,** the female members of the group during non-breeding season; **BM,** the male members of the group during breeding season; **NBM,** the male members of the group during non-breeding season.

During the breeding season, an independent T-test revealed that within-group pairwise relatedness estimates for females were significantly higher than those calculated for males [t = 2.638, df = 22, *P* (two tailed) = 0.015], though within-group pairwise relatedness estimates for females and for males were not significantly different from within-group pairwise relatedness estimates for the entire group (*P*>0.05) (Fig. 2). During the non-breeding season, an independent T-test revealed that there were no significant differences among within-group pairwise relatedness estimates for females, males or the entire group (*P*>0.05) (Fig. 2). These results indicated that within-group close relatives were more commonly female rather than male during the breeding season and that mean pairwise relatedness of males within groups was increased during the non-breeding season. An independent T-test revealed that within-group pairwise relatedness estimates for males were significantly higher during the non-breeding season relative to the breeding season [t = 2.738, df = 23, *P* (two tailed) = 0.012]; however, there were no significant differences for females or for the entire group (*P*>0.05) (Fig. 2). These results indicate that there were more close male relatives within a group during the non-breeding season.

#### B. Among-group relatedness

The number of individual pairs identified as close relatives (*r* ≥0.25) between groups was compared to analyze dispersal between groups.

**Figure 3:**
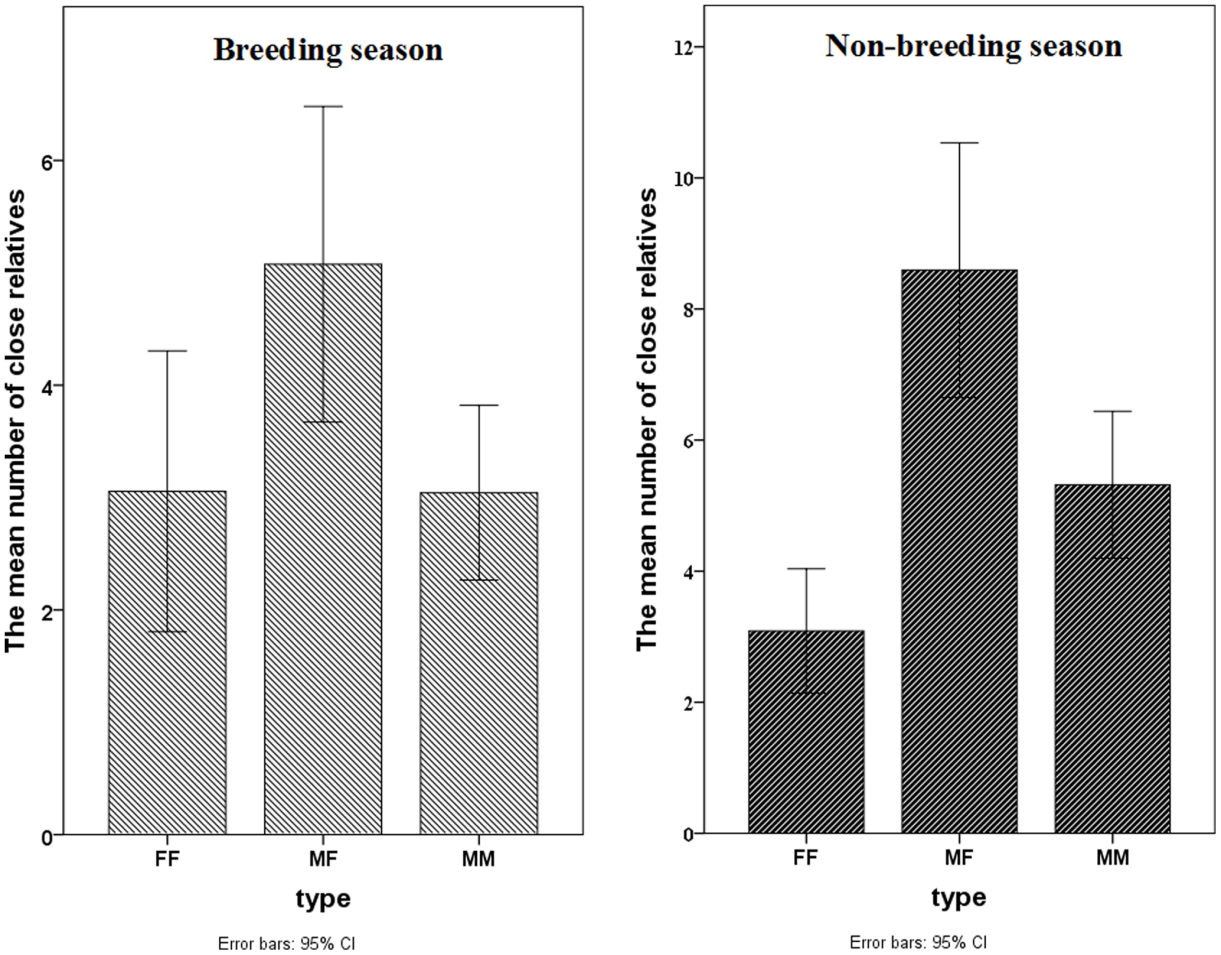
Comparison of the number of individual pairs identified as close relatives (*r* ≥0.25) among groups. **FF,** female-female pairs; **MF,** female-male pairs; **MM,** male-male pairs.

For the breeding season, a paired-sample T test revealed that the number of female-male pairs identified as close relatives was significantly greater than that of female-female pairs [paired-sample correlation = 0.776, *P*<0.0001; t = 5.413, df = 90, *P* (two tailed) <0.0001] and male-male pairs [paired-sample correlation = 0.560, *P*<0.0001; t = 4.668, df = 92, *P* (two tailed) <0.001], with no significant difference between the number of female-female pairs and male-male pairs (*P*>0.05) (Fig. 3).

For the non-breeding season, a paired-sample T test revealed that the number of female-male pairs identified as close relatives was significantly higher than that of male-male pairs [paired-sample correlation = 0.772, *P*<0.0001; t = 5.031, df = 90, t *P* (two tailed) <0.001], and that the number of male-male pairs identified as close relatives was significantly greater than that of female-female pairs [paired-sample correlation = 0.772, *P*<0.0001; t = 5.14, df = 90, *P* (two tailed) <0.001] (Fig. 3).

In general, regardless of season, the number of between-group female-male pairs identified as close relatives was the highest and the number of closely related female-female pairs was the lowest.

Together with the variation in the within-group relatedness, these results indicate male-biased dispersal. However, during the breeding season, within-group pairwise relatedness estimates for females were significantly higher than those calculated for males (*P*<0.01). This result indicates that male-biased dispersal is season-dependent.

### Seasonal Variation in Fine-scale Genetic Structure

When individuals were grouped according to the season in which they were collected, an analysis of molecular variance revealed slight but significant differences in genetic structure between the two groups (Pairwise F_ST_ = 0.017). Although there was no significant difference between females and males (Pairwise F_ST_ <0.001) during the non-breeding season, small but significant differences were observed between females and males during the breeding season, (Pairwise F_ST_ = 0.003). Consistent with genetic differentiation between the two seasonal groups, there was significant differentiation between females (Pairwise F_ST_ = 0.017) and males (Pairwise F_ST_ = 0.018) from the two seasons.

The small difference in pairwise comparison of females and males may result from dispersal during the breeding season. The fact that there was no significant difference between females and males in pairwise comparisons during the non-breeding season was most likely due to the low rate of dispersal and individual exchange during this season. The higher genetic variance observed between the two seasonal populations might be attributed to dispersal and individual exchange during the three months of sampling because all samples were collected from the same region.

## Discussion

At the beginning of the breeding season, Brandt’s vole groups are frequently composed of a single male and a female. If dispersal and individual exchanges do not occur, groups will consist of close relatives after the birth of offspring. Estimates of mean group relatedness revealed that the majority of sampled Brandt’s vole groups were comprised of closely related individuals. Mean pairwise relatedness within groups was significantly greater than zero in 85.7% (12/14) of groups sampled during the breeding season and in 92.9% (13/14) during the non-breeding season. However, only 6.38% of adult female-male pairs were identified as close relatives (including half sibs and full sibs). If random mating occurred, the expected ratio of inbreeding (the number of close-kin pairs/the sum of all possible mates) was only 6.38%. However, no actual pairings between close relatives were observed. This supports the theory that non-random mating occurs in these groups. Furthermore, the group specific F_IS_ values showed that there was no inbreeding in this natural Brandt’s vole population, suggesting that mating occurred in ways that precluded inbreeding.

A pedigree analysis performed on our natural Brandt’s vole population confirmed the polygyny found in previous studies [41]–[43]. Our results showed that the majority of breeding males were the heaviest or second heaviest individual within the groups, and that breeding females mated only with this male within groups. These results indicate that mating within a group is not random, and it may favor inbreeding avoidance by decreasing the probability of females mating with other within-group males that might be close relative. This result also provides an explanation for the observed deviation from HWE. Further parentage analysis showed that Brandt’s vole females were extra-group polyandrous, and that the majority of pregnant females had mated with one or more males outside the group, along with one within-group male. We suggest that extra-group polyandry is a reasonable explanation for the low incidence of inbreeding, because it may increase the frequency of mating among distantly-related individuals.

The mating system of this species implies a possible social hierarchy among male Brandt’s voles. The majority of reproductive males were the heaviest or second heaviest individuals in the group (Table 1). While this strategy prevents random mating within a group, the majority of males have no opportunity to copulate. Mating competition drives low-rank males to emigrate to a new breeding group and increases individual exchange between groups. While females copulate with only one male within the group, the frequent male dispersal facilitates female copulation with more distantly related out-group males.

Increased mean within-group relatedness of males and decreased genetic difference between females and males during the non-breeding season indicate that dispersal occurs mainly during the breeding season. Seasonal variation in female fine-scale genetic structures also supports the dispersal of females, though at a lower frequency than that of males. There were a greater number of between-group female-male pairs identified as close relatives than female-female pairs or male-male pairs, regardless of season. This pattern highlights the preferential separation of closely-related opposite-sex individuals, facilitated by male-biased dispersal. Thus, it appears that inbreeding avoidance during the breeding season drives male-biased dispersal in this species.

Genetic structure refers to any pattern in the genetic makeup of individuals within a population. Due to physical barriers to migration, along with a limited tendency for individuals to move or spread (vagility), and a tendency to remain or come back to their natal place (philopatry), natural populations rarely all interbreed as assumed in theoretical random models (panmixy) [56]. The genetic structure and demography of local populations are tightly linked to the rate and scale of dispersal [36]. The fact that the temporal variance in genetic structure was consistent with the season-dependent dispersal in this species supports this hypothesis. The season-dependent male-biased dispersal is not only the basis of the polygynous and extra-group polyandrous mating strategy in this species but also caused the fine variance in genetic structure between females and males as well as the temporal variance of genetic structure between populations from different seasons.

Many animals have evolved mechanisms that prevent them from breeding with close relatives. Inbreeding avoidance has commonly been used to interpret social behaviors such as reproduction skew, individual dispersal, and construction of the mating system. However, ecological constraints are ultimately believed to provide the selective pressure for these reproductive and behavioral differences, and the levels of inbreeding depression vary across taxa, populations and environments [3], [57]. In stringent and severe environments, inbreeding can become necessary for the survival and persistence of a group. Cheptou and Donohue (2011) indicated that inbreeding depression is environment-dependent, which often has important ecological and evolutionary consequences. Inbreeding depression in some environments may even contribute to adaptation in others [1]. Inbreeding has been described as a derived trait in the naked mole-rat, *Heterocephalus glaber*, and might have evolved as an adaptive response to the high costs of dispersal [57]. Inbreeding is tolerable and common in Brandt’s voles under laboratory conditions, and some ecologists have suggested that the mating system changes with habitat. Wang reported evidence of inbreeding in this species [58], though 3 of 7 microsatellite markers used in their analysis had a high frequency of null alleles, possibly indicating a problem in their analysis. However, the discrepancy with their results might be caused by the different sampling conditions. Additional studies should therefore be conducted to obtain more detailed information regarding variations among groups and social structures throughout a complete annual cycle under different ecological habitats. Such studies will help us to better understand the mechanism of inbreeding avoidance in this species.

In conclusion, the levels of genetic diversity within our natural Brandt’s vole population were affected by seasonal changes in group structure and seasonal reproduction. We conclude that inbreeding avoidance during the breeding season drives the polygynous and extra-group polyandrous mating system that determines the fine-scale genetic structure.

## Materials and Methods

### Sampling Design and Animal Capture

To assess the genetic and social structure of Brandt’s vole populations over the breeding and non-breeding seasons, a total of 249 individuals were captured from 15 groups during the breeding season, and a total of 200 individuals were captured from 14 groups during the non-breeding season. A summary of the group structures is provided in Table 1. Trapping plots were established in a typical steppe near the town of Aershanbaolige in Xinlingol (N44°40′, E115°40′), Inner Mongolia, China (Fig. S1), which is a typical habitat for Brandt’s voles. Voles were captured on June 18, 2007, for the breeding season analysis. This season was predicted to show the most complex group structure because of sexual maturity, dispersal, and individual exchange. For the non-breeding season analysis, voles were captured on September 28, 2007 This season was predicted to exhibit the most stable group structure because dispersal and individual exchange were complete and mating competition had ended [39], [40]. All of the individuals in every group were captured by snap-trapping using peanuts as bait. To ensure that all of the individuals were captured, snap-traps were set at all of the group’s burrow entrances, and trapping continued until individuals had stopped merging. The burrow entrances were then blocked with soil and checked the following day to determine whether any individuals entered or exited the group. Each animal captured was weighed and sexed, after which tissue samples were collected from the foot and stored in 75% ethanol until analysis. Embryos of pregnant females large enough to rule out contamination with maternal tissue were carefully separated and stored in 75% ethanol for paternity analysis. Our trapping and handling of Brandt’s voles in the field was approved by the Institutional Animal Use and Care Committee of the Institute of Plant Protection, Chinese Academy of Agricultural Sciences.

### Microsatellite Analysis

Genomic DNA was extracted from tissue samples using the standard proteinase K, phenol, and chloroform protocol [59]. Genetic variation was examined at 14 microsatellite loci that had previously been identified in Brandt’s voles [53], [54]. Characteristics of the microsatellite markers are summarized in Table S1. One primer of each of the 14 pairs was end-labeled with either FAM or HEX fluorescent dyes (Sangon). The PCRs were performed in 15 µL reaction volumes containing 200 µM of each dNTP, 8 mM Tris-HCl (pH 8.3), 40 mM KCl, 1.2 mM MgCl2, 0.4 µM of each primer, 80 ng of genomic DNA template and 0.25 U Golden DNA Polymerase (TIANGEN). The amplification profiles consisted of initial denaturation at 94°C for 5 min, 35 cycles of 94°C for 30 s, 45 s at the annealing temperature (see T_a_ in Table 1), and 72°C for 80 s, and a final extension at 72°C for 5 min. The PCR products were then diluted to 50 ng/µL, after which 1-µL of each diluted PCR products was mixed with 1.2 µL deionized formamide and 0.2 µL GeneScan350 ROXTM or GeneScan-500 ROXTM (ABI) internal standard and run on a 377 genetic analyzer (ABI). The fragment sizes of the PCR products were analyzed using GeneScan 3.7 and GeneMarker 1.75. PCR mixtures with known allele sizes were also added to each acrylamide gel as another standard. The program MICRO-CHECKER 2.0 [60] was used to test for the presence of null alleles, short dominance, and typing errors. The Cervus 3.0 software [61] was used to calculate the number of alleles, the frequency of null alleles, observed heterozygosities, expected heterozygosities, and polymorphic information content (PIC). Genalex 6.1 (Peakall and Smouse 2006) was used to test Hardy–Weinberg equilibrium (HWE).

### Group Pedigree Reconstruction and Mating System Inference

The relatedness of individuals within groups was inferred using COLONY version 2.0, which is based on the maximum-likelihood (ML) method. For this analysis, we set the typing error rate at the suggested 0.025 [62], [63]. The male mating system and female mating system were both specified as polygamous, and all other parameters were set as default. Individuals whi weighed more than 10 g after the removal of viscera were considered physiologically mature [64] and were used for data analysis. Only the inferred fathers, mothers, full sib pairs and half sib pairs that had a probability of ≥0.95 (as determined by the software) were included in the statistical analysis. During the breeding season, all of the non-adults, including all of the embryos that were developed enough to be separated clearly from the uterus, were used for group pedigree reconstruction. However, only adults were used for comparison of group structures during the breeding season. Paternity of the embryos was inferred using COLONY version 2.0.

### Statistical Analysis

Unless stated otherwise, all analyses were performed using Genalex 6.1 [65] and SPSS 15.0 for Windows (SPSS Inc). The means, plus or minus one standard deviation, are reported, unless otherwise noted.

#### Inbreeding detection

Group specific F_IS_ values were estimated using the Arlequin 3.11 software [66] to detect signs of inbreeding. Statistical significance was tested by1000 random permutations. To avoid the possible influences of emigrated and immigrated group members, only embryos and juveniles were used to estimate the group-specific F_IS_ values.

#### Individual dispersal and group member exchange inference

Individual dispersal and recombination were inferred by analyzing the relatedness of individuals from different groups. Pairwise relatedness estimates devised by Queller and Goodnight [67] were calculated for each pair of individuals, and mean within-group pairwise relatedness for each group was estimated at the 95% confidence interval via bootstrapping. Random permutation of the dataset was used to generate a distribution for the null hypothesis of no relatedness among individuals within groups and to provide a test for significance. All bootstrapping and permutational tests were performed 1000 times. Mean within-group pairwise relatedness for each sex in each group was estimated and compared with the total within-group pairwise relatedness of each group.

Close relative pairs (individual pairs with *r* ≥0.25) between every two groups, including female-female pairs, female-male pairs and male-male pairs, were calculated separately and compared to each other using one-way analysis of variance (ANOVA) in order to detect the evidence of sex-biased dispersal.

#### Analyses and comparison of genetic structure

An analysis of molecular variance (AMOVA) was used to estimate the group pairwise FSTs. These group pairwise FSTs were estimated separately for each sex in order to detect evidence of sex-biased dispersal. Statistical significance was tested by 1000 random permutations. Individuals collected during the same season were treated as a population, and AMOVA was used to estimate population pairwise FSTs and the partitioning of genetic variation within and between seasonal populations.

## Supporting Information

Figure S1 **Geographical positions of sampled Brandt’s vole groups by GPS in Inner Mongolia, China.** Groups with blue flags have been sampled on June 18^th^, 2007 for breeding season, and groups with red flags have been sampled on September 28^th^, 2007 for non-breeding season.
(TIF)

Table S1 **Summary statistics for 19 microsatellite loci of** ***Lasiopodomys brandtii*** **.**
(DOC)

Table S2 **Summary of HWE.**
(XLS)
