# Supplementary figures and images for: Inbreeding Avoidance Drives Consistent Variation of Fine-Scale Genetic Structure Caused by Dispersal in the Seasonal Mating System of Brandt’s Voles
Source: PLoS One. 2013 Mar 14;8(3):e58101. doi: 10.1371/journal.pone.0058101 (PMC3597616; doi:10.1371/journal.pone.0058101)

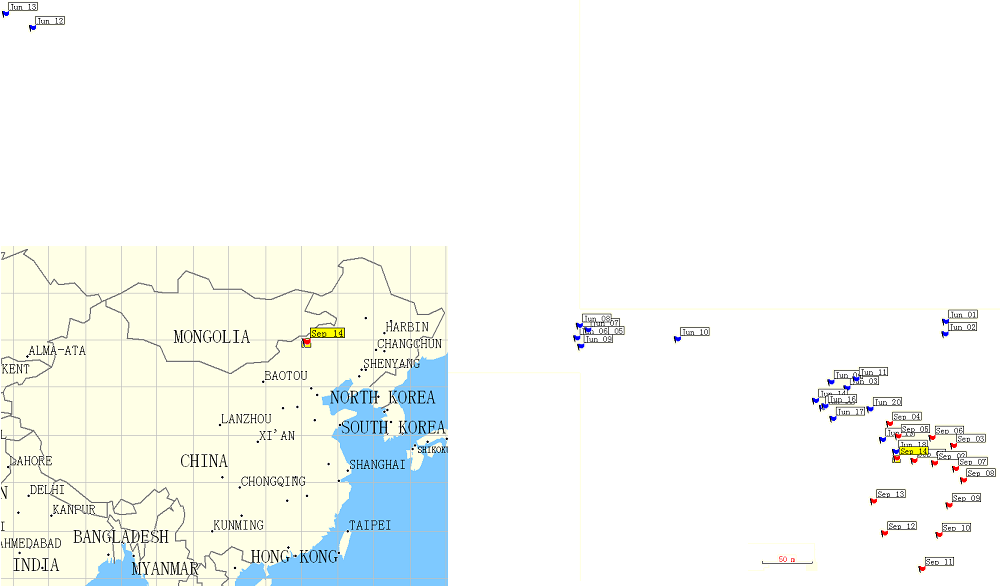

Supplement: Figure S1 — Geographical positions of sampled Brandt’s vole groups by GPS in Inner Mongolia, China. Groups with blue flags have been sampled on June 18th, 2007 for breeding season, and groups with red flags have been sampled on September 28th, 2007 for non-breeding season. (TIF) [file pone.0058101.s001.tif]
