# Supplementary material for: Inbreeding Avoidance Drives Consistent Variation of Fine-Scale Genetic Structure Caused by Dispersal in the Seasonal Mating System of Brandt’s Voles
Source: PLoS One. 2013 Mar 14;8(3):e58101. doi: 10.1371/journal.pone.0058101 (PMC3597616; doi:10.1371/journal.pone.0058101)
Supplement: Table S1 — Summary statistics for 19 microsatellite loci of Lasiopodomys brandtii . (DOC) [file pone.0058101.s002.doc]

*Table S1 Summary statistics for 19 microsatellite loci of Lasiopodomys brandtii*

| Locus | Size of the cloned allele (bp) | Ta(℃) | K | N | Ho | He | PIC | F (null) |
| --- | --- | --- | --- | --- | --- | --- | --- | --- |
| BVM01 | 161-185 | 58 | 558 | 8 | 0.480 | 0.483 | 0.462 | -0.0087 |
| BVM08 | 220-240 | 64 | 549 | 2 | 0.505 | 0.477 | 0.363 | -0.0282 |
| BVM03 | 154-188 | 64 | 529 | 17 | 0.781 | 0.833 | 0.813 | 0.0327 |
| BVM02 | 142-160 | 58 | 519 | 9 | 0.771 | 0.803 | 0.774 | 0.0216 |
| BVM04 | 170-196 | 55 | 531 | 10 | 0.674 | 0.699 | 0.658 | 0.0128 |
| BVM09 | 167-210 | 58 | 518 | 7 | 0.602 | 0.592 | 0.546 | -0.0171 |
| BVM05 | 167-197 | 55 | 492 | 12 | 0.695 | 0.676 | 0.625 | -0.0154 |
| BVM11 | 163-205 | 61 | 429 | 21 | 0.874 | 0.891 | 0.880 | 0.0088 |
| Mb29n | 222-254 | 66 | 435 | 11 | 0.736 | 0.779 | 0.751 | 0.0321 |
| Mb13n | 294-308 | 64 | 503 | 8 | 0.757 | 0.789 | 0.755 | 0.0202 |
| Mb11n | 184-194 | 64 | 519 | 5 | 0.697 | 0.693 | 0.639 | -0.0012 |
| Mb28n | 248-278 | 63 | 532 | 16 | 0.821 | 0.829 | 0.808 | 0.0028 |
| Mb03n | 317-331 | 62 | 529 | 9 | 0.781 | 0.839 | 0.817 | 0.0362 |
| Mb24n | 307-342 | 66 | 479 | 14 | 0.808 | 0.793 | 0.765 | -0.0106 |
| Mean | --------- | ------ | ------ | 10.64 | 0.713 | 0.727 | 0.690 | ------ |

Ta(°C), the optimal annealing temperature; K, the number of individuals; N, the number of alleles per locus; Ho, the observed heterozygosity; He, the expected heterozygosity; PIC, polymorphism information content; Key: NS = not significant, * = significant at the 5% level, ** = significant at the 1% level, *** = significant at the 0.1% level, ND = not done; F (null), the frequency of null alleles.
